# Sociodemographic and health factors associated with the risk of financial catastrophe when informal care for patients with haematological neoplasms is replaced by formal care

**DOI:** 10.1186/s13561-022-00364-0

**Published:** 2022-03-09

**Authors:** Raúl del Pozo-Rubio, Marta Ortega-Ortega

**Affiliations:** 1 Department of Economics and Finance, Faculty of Social Sciences, University of Castilla-La Mancha, Avenida de los Alfares, 44. C.P.: 16.071, Cuenca, Spain; 2 Department of Applied and Public Economics, and Political Economy, Complutense University of Madrid, Campus de Somosaguas s/n. 28.023, Madrid, Pozuelo de Alarcón, Spain

**Keywords:** Informal care, Formal care, Haematologic neoplasms, Financial catastrophe, D63, I0, I38

## Abstract

**Background:**

Cancer is one of the diseases with the highest incidence and mortality in the world, and one that requires greater care (formal and informal). At present, the traditional informal caregiver is disappearing. The objective is to analyse the sociodemographic and health factors associated with the possible catastrophic financial effect on households of replacing informal care by formal care for patients with blood cancer, during the different stages of treatment in Spain.

**Methods:**

A total of 139 patients with haematological neoplasm who underwent stem cell transplantation completed a longitudinal questionnaire during each of three treatment phases. Of this population, 88.49% received informal care. The households were classified into those where the replacement of informal care with formal care would impose a financial burden exceeding 40% of equivalent household income, versus those who would not suffer this consequence. Three logistic regression models (one for each treatment phase) were estimated and the corresponding marginal effects determined.

**Results:**

The factors associated with a higher probability of financial catastrophe were married marital status, low education level, fair to very poor self-perceived health status, the diagnosis of leukaemia in the pre-transplant and first-year post-transplant phases and of multiple myeloma disease in the final post-transplant phase.

**Conclusions:**

These findings reveal the need to design social policies to meet the care needs of patients with blood cancer which at present are covered by informal care. Given the foreseeable elimination of this option, these families must be protected from the financial burden incurred from the use of privately-contracted assistance.

## Background

According to the World Health Organization, cancer, cardiovascular diseases and AIDS will soon be the three diseases making the greatest impact on society, in terms of incidence, limitation of daily life and mortality [1]. Currently, cancer is the second cause of death worldwide, responsible for one in every six deaths [1], while in countries like Spain it is already the first cause of death, responsible for one in four deaths [2].

In 2018, 18.1 million new cases of cancer and 9.6 million deaths from this disease were detected worldwide [3]. Of these figures, 3.9 million new cases (21.5%) and 1.9 million deaths (19.8%) took place in Europe, although the continent has only 9% of the world’s population [4]. Today, the incidence of cancer and the corresponding mortality rates worldwide are 20.20 and 10.63%, respectively [3]. During their lifetimes, half of all men and a third of women are expected to suffer from cancer [5], one of the most complex diseases facing society today.

In 2018, the cancer with the highest incidence worldwide was lung cancer (11.6% of total cases) followed by female breast cancer (11.6%) and prostate cancer (7.1%). The highest levels of mortality were recorded for lung cancer (18.4% of all deaths caused by cancer), followed by colorectal cancer (9.2%), stomach cancer and liver cancer (8.2%). In fourth and fifth place, for incidence and mortality, respectively, was the group of haematological neoplasms, or blood cancer (Hodgkin/non-Hodgkin lymphoma, leukaemia and multiple myeloma), of which there were 1.19 million cases in 2018 with an incidence of 6.56%, producing 0.7 million deaths (a mortality rate of 7.22%) [3]. The incidence and mortality of these forms of cancer are expected to have increased sharply worldwide (by 55.93 and 65.49%, respectively) by the year 2040 [6]. In the case of Spain (population 46.7 million), these percentages are expected to increase less than at the global level for haematological neoplasm to the percentages of 8.06% for incidence and 8.29% for mortality in 2040, assuming an increase compared to 2018 of 22.09 and 30.09%, respectively [6].

Aggressive cancer treatment can provoke multiple side effects, such as nausea and vomiting [7], fatigue [8, 9], loss of appetite and weight [10], hair loss [11], pain [12] and depression and anxiety [13, 14], affecting both social and work-related activities [15]. These consequences drastically restrict personal daily activity, meaning that in most cases the assistance of a caregiver is required in order to perform basic activities of daily living [16, 17]. As shown in a recent study, this treatment impact can be so severe that the chemotherapy administered at the end of life not only does not produce an improvement in the patient’s quality of life, but reduces it, even in patients with good performance status [18].

For haematological neoplasms, and depending on the patient’s clinical situation, the indicated treatments are chemotherapy, radiation therapy, supportive therapy, targeted therapy and haematopoietic stem cell transplantation (HSCT) [19]. The latter therapy is considered one of the most appropriate and effective treatments for more malignant prognoses [20]. The main advantages of HSCT are the compression of disability, which facilitates the provision of more intensive treatment, plus longer life expectancy and increased tolerable immunosuppression, which reduces toxicity [21]. However, HSCT patients are among the most vulnerable and acutely ill of all cancer populations [22, 23], due to the numerous complications associated with HCST itself, such as the timeline of infections, sinusoidal obstruction syndrome, other gastrointestinal and liver diseases [24] and, with especial severity, graft vs. host disease [25].

Two types of care may be provided for cancer patients: formal and informal. Formal care refers to services that are contracted and paid for, and provided by care professionals working either for public institutions or in the private sector [26, 27]. This form of care provision may be home-based, community-based (via day/night care centres) or residential [26, 28]. Informal care, on the other hand, is a nonmarket commodity [29], usually unpaid [30, 31] and provided voluntarily [31], often by family members in the patient’s immediate social environment [29], or by other relatives or friends [32]. This informal care covers a wide range of activities, and may include activities of daily living (ADLs), instrumental activities of daily living (IADLs) and supervision [33], in areas such as basic care, assistance with cleaning, shopping, financial matters, etc., [34].

The needs of persons with cancer can be so intense and constant that informal caregivers must reduce or give up their working hours, and hence suffer a significant drop in income [35], in order to dedicate time to assist with medical consultation, hospitalisation or care in the home [36]. Moreover, their leisure time is reduced, and in many cases their own health and quality of life are seriously affected [37–44].

In both types of care, a wide range of tasks are performed. However, informal caregivers rarely receive training or instruction in this respect, in contrast to formal caregivers, although in the latter case, too, the level and intensity of training received may vary considerably [26]. Various theories have been proposed to analyse the use and needs of each type of care, whether alone or in combination with the other. The theory of supplementary care postulates that most such responsibilities are discharged by means of informal care, while formal care is only resorted to temporarily or circumstantially [45–47]. The theory of complementary care goes a step further, arguing that formal care is employed when the patient’s needs exceed the skills and capacities of informal care [46, 47]. Finally, and more specifically, the theory of the hierarchical compensatory model establishes a ranking of preferences for the person who is to provide informal care (first the spouse, then the children or parents, then other caregivers), and only when this person is not available does formal care appear [46–48].

In today’s society, with the increasing presence in the job market of women, who have historically played a leading role in providing informal care [49], and the changes that have taken place in family structures [50], the figure of the traditional informal caregiver is disappearing, and the demand for professional services is rising sharply [51–54]. In the case of Spain, the services related to home care must be financed privately (by the patients and/or their families), publicly, or by a combination of the two sources, if informal care is not available, because the rest of health expenditure (oncological treatments (or other treatments needed), medical tests, hospitalizations, consultations, medications, etc.) is financed by the Spanish National Health System. Furthermore, in the case of patients with cancer, as the severity of the patient’s condition increases, informal care is more likely to be replaced by formal care [55].

A recent study revealed that over 75% of patients diagnosed with blood cancer receive informal care [56], and that in many cases these informal caregivers play the role of advocate, protector and/or symptom monitor [57]. Other researchers have analysed the impact that would be produced on household finances if informal care had to be replaced by formal care for patients with haematologic neoplasm. This study concluded that over 80% of families would have to dedicate six times their monthly income to be able to attend to the needs of cancer patients during the provision of chemotherapy and/or radiotherapy, and five times their monthly income from the first to the sixth year after HCST [58]. To our knowledge, however, no previous study has been undertaken to examine the sociodemographic and health profiles of those liable to be affected by this financial burden, and associated factors.

In view of the above considerations, our study aim is to analyse the sociodemographic and health factors associated with the financial catastrophe that may be provoked by the replacement of informal care by formal care for patients with cancer. As informal caregivers do not receive financial compensation for performing these tasks, while formal care must be paid for, our working hypothesis is that of the perfect substitution of informal care by formal care.

## Methods

The present study is based on the results obtained in previous research into the catastrophic financial effects experienced following the replacement of informal care by formal care for patients diagnosed with haematological neoplasms [58]. The basis for the earlier study was a descriptive, longitudinal questionnaire, designed to obtain socio-demographic and clinical information about cancer patients and their primary informal caregivers during different phases of treatment [59]. Eligible patients included adults (≥16 years) diagnosed with any type of haematological neoplasm, such as acute leukaemia, Hodgkin lymphoma, non-Hodgkin lymphoma, multiple myeloma, and other less common malignant haematological diseases, who had undergone stem cell transplantation between 2006 and 2011 at the two reference hospitals for malignant haematological diseases in the south of Spain (University Hospital Virgen de las Nieves in Granada and the University Hospital Virgen del Rocio in Seville), and at the time of the survey, had survived the disease. The Clinical Research Ethics Committee and Haematology Department of each health centre approved the study.

The information was collected between January 2012 and December 2013. All of the eligible patients (*n* = 299) were contacted by telephone. Patients that randomly (*n* = 230) responded to the first or second call, were informed and invited to participate in the study by sending a questionnaire by mail. The patients completed the questionnaire, providing information about their socio-demographic and clinical characteristics. In addition, they were asked to identify their primary informal caregiver during their illness and to answer the questions related to the primary caregiver and the number of hours and months of informal care they received from him or her.

In the present study, three sequential phases of treatment evolution are defined, reflecting the medical protocol applied [60]. The first, or pre-transplant, phase, is defined as the period between the initial point of the treatment protocol and the moment when stem cell transplantation is performed. The prior administration of cycles of chemotherapy and/or radiotherapy is included in this phase. The second phase covers the first year after the transplant, a period during which the patient is especially vulnerable, requiring in-hospital isolation for the infusion of the haematopoietic stem cells and exhaustive medical control. The complexity of this process makes the recovery phase especially delicate. Finally, the third phase lasts from the second to the sixth years after the transplant, and includes the total period of recovery. This may be prolonged and is characterised by the patient’s special vulnerability until complete remission is achieved.

The informal care value (ICV) is obtained according to the number of hours of informal care required in each of the above phases [61] and is used to estimate the price of replacement formal care. The catastrophic financial effects of this replacement have been described in a previous study [58].

Both of the above studies used the three traditional methods for assessing informal care, i.e., opportunity cost, proxy good and contingent valuation [62]. In the present study, in view of the widely varying estimates obtained in each of the three treatment phases, and in order to simplify the analysis, but at the same time take associated factors into consideration, we decided to use the proxy good method. This method considers each hour of informal care provided at the price that would be charged by a professional performing the same care and attention tasks as the informal caregiver [29, 61]. Therefore, it is the most appropriate method to assess the replacement of informal care by formal care. In this point, the Spanish Dependency Act [63], which offers a complete and varied catalogue of services to attend to people who need permanent care, and establishes two prices per hour for home care service. If the hours provided by the professional carer are dedicated to personal care, the price is €14 per hour, while for hours dedicated to household activities, the price is €9 per hour. In addition to these two prices that would form an interval, we have estimated the mean, assuming a scenario where half of the hours are dedicated to the personal care of a patient with haematological neoplasm, and the other half of the hours of informal care are dedicated to household activities. Therefore, the final price used is €11.50 per hour [61].

Accordingly, and following other indications in the literature, the households were classified into two groups: those that, if informal care were replaced by formal care, would have to dedicate more than 40% of their equivalent income to such care (ICV > 40% household equivalent income); and those which would need to dedicate less than 40% of their equivalent income to this purpose. For these calculations, we used the measure of financial catastrophe defined by Wagstaff and Van Doorslaer (2003) [64]. The understanding behind this definition is that out-of-pocket (OOP) payments for replacing informal care by formal care (ICV) would provoke a catastrophic increase in expenditure for households if the resulting co-payments led to a significant decrease in the household’s standard of living [65, 66]. The threshold for this catastrophe is defined as the percentage of household income that would have to be dedicated to making the corresponding OOP payment for formal care in replacing informal care.

In accordance with our study aims, and in line with previous work in this field [67–72], we ran three binary logistic regression models, reflecting the binary nature of the dependent variable, one for each of the three time periods analysed (yi = 1, if ICV exceeds 40% of household income, yi = 0 if ICV does not exceed 40% of household income, with i = 1,..,n, where n is the number of individuals in the sample). The specification of the models is as follows [73]:

1 $$ {y}^{\ast }={X}^{\prime}\beta +\varepsilon $$

where y* is not observed, X represents the matrix of explanatory variables, ß is a vector of the parameters and ε is the standard error following logistic probability distribution. In addition, for the binary model:

$$ y=0\leftrightarrow {y}^{\ast}\le \theta $$

2 $$ y=1\leftrightarrow {y}^{\ast }>\theta $$

where θ refers to the parameter assigned to each of the two categories in the dependent variable for financial catastrophe.

The above models were used to analyse the sociodemographic and health characteristics that present statistically significant parameters, and therefore are associated with the corresponding dependent variables, adjusted for all other features. Marginal effects were estimated for all the variables.

Taking previous work in this field into consideration, the following explanatory variables, essentially sociodemographic characteristics, were selected [67–72] (the model reference variable is indicated by *): gender (male; female*); age (below 35*; 35–44; 45–54; over 55); marital status (married*; single; widowed; separated/divorced); education level (low level*: illiterate/primary school incomplete/primary or equivalent; middle level: secondary school; high level: university degree or equivalent); loss of employment (vs. remain employed); diagnosis (acute leukaemia*; Hodgkin lymphoma/non-Hodgkin lymphoma; multiple myeloma; other); self-perceived health status (very poor/poor/fair *; good/very good). The self-perceived health status is rated by the patient on a Likert-type scale (very poor health status; poor health status; fair health status; good health status; very good health status).

Household income was not included due to the small sample size; moreover, there were problems of multicollinearity with marital status and with the respondent’s education background.

All statistical analyses were performed using the Stata 16.0 package (StataCorp LP, College Station, TX).

## Results

In our study sample, 123 patients reported having received informal care at some time during their treatment (88.49% of survey respondents). Table 1 shows the sociodemographic information for the sample, according to static and dynamic variables. Among the former, half of the patients were female (48.78%), with an average age of 46.42 years (SD: 13.93). The majority were married (65.85%) and the largest group had a low level of education (primary school incomplete, primary or equivalent) (37.40%). Regarding the clinical variables for type of cancer, a slightly higher incidence of lymphoma was observed (32.52%), followed by multiple myeloma (29.27%). Among the dynamic variables, 20.33, 39.02 and 62.60% of the patients stated they had enjoyed good or very good self-perceived health status during the pre-transplant, first-year post-transplant and second-to-sixth-year post-transplant periods, respectivelyThe numbers of patients in employment fell considerably during the treatment phases (71.22, 31.66 and 24.46% in the pre-transplant, first-year post-transplant and second-to-sixth-year post-transplant period, respectively), whilst those of retirees or persons receiving an earnings-related pension rose during the same periods (6.47%, 35.97 and 50.36%, respectively). The average number of hours received in the pre-transplant, first-year post-transplantation and second-to-sixth-year transplantation phase amounts to 233.66 (SD: 141.30), 190.98 (SD: 145.11), 58.54 (SD: 101.98) monthly hours, respectively. Therefore, the average economic valuation of the hours of informal care received during the pre-transplantation, transplantation and post-transplantation phases using the proxy good method with the price of 11.5 €/hour increases to €2800.93 (SD: €143.55), €2289.28 (SD: €150.96) and €549.15 (SD: € 85.67) per month, respectively.

**Table 1:** Sociodemographics and health characteristics of patients

*Static variables*	%		
Gender			
Male	51.22		
Female	48.78		
Age (Mean (SD); Mín-Máx)	46.42 (13.93); 17–67		
Marital Status			
Married	65.85		
Single	24.39		
Widow	2.44		
Separated/divorced	7.32		
Educational level			
Low level (primary school incomplete, primary or equivalent)	37.40		
Middle level (secondary school/ middle level professional)	33.33		
High (University degree or equivalent)	29.27		
Diagnosis			
Acute leukemia	26.02		
Hodgkin lymphoma/No Hodgkin lymphoma	32.52		
Multiple mieloma	29.27		
Other	12.20		
*Dinamic variables*			
	Stage 1: Pretrasplantation	Stage 2: 1° year after trasplantation	Stage 3: 2°-6° year after trasplantation
Self-perceived health status			
Very bad/bad/regular	79,67	60,98	37,40
Good / very good	20,33	39,02	62,60
Work status			
Employed (Employed for others, autonomous)	69,92%	27,64%	21,14%
Unemployed	5,69%	12,20%	10,57%
Receiving earnings-related pension	6,50%	38,21%	52,03%
Other situations (Housework, student, other)	17,89%	21,95%	16,26%
Monthly household income (€) (Mean (SD))	1732.11 (1192.28)	1683.33 (1088.01)	1801.22 (1220.19)
Monthly informal care hours received (Mean (SD))	233.66 (141.30)	190.98 (145.11)	58.54 (101.98)
Value of informal care (Monthly €)			
Proxy Good Method (9 €/h) (Mean (SD))	2192.03 (112.35)	1791.61 (118.14)	701.69 (109.46)
Proxy Good Method (11.5 €/h) (Mean (SD))	2800.93 (143.55)	2289.28 (150.96)	549.15 (85.67)
Proxy Good Method (14 €/h) (Mean (SD))	3409.83 (174.76)	2786.95 (183.78)	854.24 (133.26)

Note: *SD* Standard deviation

Tables 2, 3 and 4 show the marginal effects calculated from each binary logistic regression concerning the catastrophic financial effect produced by replacing informal care by formal care during each of the study phases. In the first phase (pre-transplant) (Table 2), the sociodemographic determinants significantly associated with a reduced probability of financial catastrophe were age 35–44 years (− 22.17%); widowhood vs. married status (− 44.77%); and having a secondary school education (− 16.76%) or a university education (− 21.95%), vs. a low level of education. In terms of clinical diagnosis, multiple myeloma and other forms of blood cancer were associated with a lower probability of catastrophe, with respect to leukaemia (− 27.85% and − 33.23%, respectively). Finally, patients who had a good or very good self-perceived health status were 21.50% less likely to suffer financial catastrophe than those with very poor, poor or fair self-perceived health status.

**Table 2:** Marginal effects for the binary logistic regression model performed for the catastrophic measure. Pre-transplant or first phase

		dy/dx	SD	*P*-value
Male (Ref. Female)		1.69%	0.063	0.788
Age (Ref. Age < 35)	35–44	−22.17%	0.101	0.029**
	45–54	−4.30%	0.109	0.694
	> 55	2.77%	0.115	0.810
Marital Status (Ref. Married)	Single	8.45%	0.085	0.322
	Widow	−44.77%	0.125	0,000***
	Separated/Divorced	14.67%	0.141	0.298
Educational level (Ref. Low level: primary school incomplete, primary or equivalent)	Middle level: secondary school/middle level profesional	−16.76%	0.085	0,048**
	High level: University degree or equivalent	−21.95%	0.072	0.000***
Activity Status (Ref. Employed (employed for others, autonomous))	Unemployed	−13.48%	0.109	0.216
	Receiving earnings-related pension	13.39%	0.121	0.072*
	Other situations (Housework, student, other)	6.71%	0.103	0.513
Diagnosis (Ref. Acute Leukaemia)	Hodgkin Lymphoma/Non Hodgkin Lymphoma	−10.48%	0.109	0.314
	Multiple Myeloma	−27.85%	0.119	0.014**
	Other	−33.23%	0.132	0,006***
Self-perceived health status (Ref. Very bad/bad/regular self-perceived health status)	Good/very good self-perceived health status	−21.50%	0.074	0,004***
N	123			
LR χ^2^ (H_0_: β_1_ = β_2_ = … = β_k_)	36.80			
Prob > χ^2^	0.000			
Pseudo R^2^	0.390			
Classification table	89.17%			

*dy/dx*: Marginal effect. Includes the slope of the calculated parameter

*SD* Standard deviation

*p-value*: Corresponds to the test of individual significance of the corresponding parameter

*LR*: Corresponds to the test of overall significance of all the slopes in the model

*** Denotes significance at the level of *p* < 0.01; ** denotes significance at the level of *p* < 0.05; * denotes significance at the level of *p* < 0.10

**Table 3:** Marginal effects for the binary logistic regression model performed for the catastrophic measure. First year post-transplant or second phase

		dy/dx	SD	*P*-value
Male (Ref. Female)		−11.52%	0,067	0,086*
Age (Ref. Age < 35)	35–44	−4.16%	0.098	0.672
	45–54	−3.43%	0.103	0.738
	> 55	−2.48%	0,103	0.809
Marital Status (Ref. Married)	Single	11.52%	0.089	0.192
	Widow	−23.00%	0.159	0.148
	Separated/Divorced	−5.89%	0.102	0.568
Educational level (Ref. Low level: primary school incomplete, primary or equivalent)	Middle level: secondary school/middle level profesional	−13.57%	0.083	0.102
	High level: University degree or equivalent	−20.39%	0.078	0.009***
Activity Status (Ref. Employed (employed for others, autonomous))	Unemployed	−8.12%	0.106	0.442
	Receiving earnings-related pension	4.66%	0.075	0.533
	Other situations (Housework, student, other)	−3.31%	0.102	0.745
Diagnosis (Ref. Acute Leukaemia)	Hodgkin Lymphoma/Non Hodgkin Lymphoma	15.07%	0.082	0.066*
	Multiple Myeloma	−1.15%	0.082	0.888
	Other	−7.49%	0,095	0.428
Self-perceived health status (Ref. Very bad/bad/regular self-perceived health status)	Good/very good self-perceived health status	−24.55%	0,062	0,000***
N	123			
LR χ^2^ (H_0_: β_1_ = β_2_ = … = β_k_)	32.51			
Prob > χ^2^	0.000			
Pseudo R^2^	0.275			
Classification table	88.49%			

*dy/dx*: Marginal effect. Includes the slope of the calculated parameter

*SD* Standard deviation

*p-value*: Corresponds to the test of individual significance of the corresponding parameter

*LR*: Corresponds to the test of overall significance of all the slopes in the model

*** Denotes significance at the level of *p* < 0.01; ** denotes significance at the level of *p* < 0.05; * denotes significance at the level of *p* < 0.10

**Table 4:** Marginal effects for the binary logistic regression model performed for the catastrophic measure. Second-sixth year post-transplant or third phase

		dy/dx	SD	*P*-value
Male (Ref. Female)		2.54%	0,080	0.751
Age (Ref. Age < 35)	35–44	4.75%	0.137	0.729
	45–54	14.98%	0.126	0.234
	> 55	15.39%	0.136	0.258
Marital Status (Ref. Married)	Single	18.10%	0.108	0.092*
	Widow	−11.50%	0.249	0.644
	Separated/Divorced	−13.19%	0.174	0.449
Educational level (Ref. Low level: primary school incomplete, primary or equivalent)	Middle level: secondary school/middle level profesional	−12.03%	0.097	0.216
	High level: University degree or equivalent	−8.59%	0.105	0.413
Activity Status (Ref. Employed (employed for others, autonomous))	Unemployed	14.05%	0.148	0.343
	Receiving earnings-related pension	10.04%	0.106	0.925
	Other situations (Housework, student, other)	4.82%	0.136	0.723
Diagnosis (Ref. Acute Leukaemia)	Hodgkin Lymphoma/Non Hodgkin Lymphoma	15.32%	0.101	0.127
	Multiple Myeloma	23.39%	0.110	0,003**
	Other	0.90%	0.137	0.948
Self-perceived health status (Ref. Very bad/bad/regular self-perceived health status)	Good/very good self-perceived health status	−42.18%	0.081	0,000***
N	123			
LR χ^2^ (H_0_: β_1_ = β_2_ = … = β_k_)	40.67			
Prob > χ^2^	0.000			
Pseudo R^2^	0.214			
Classification table	74.82%			

*dy/dx*: Marginal effect. Includes the slope of the calculated parameter

*SD* Standard deviation

*p-value*: Corresponds to the test of individual significance of the corresponding parameter

*LR*: Corresponds to the test of overall significance of all the slopes in the model

*** Denotes significance at the level of *p* < 0.01; ** denotes significance at the level of *p* < 0.05; * denotes significance at the level of *p* < 0.10

Table 3 shows the results for phase 2. The variables high education level and good/very good self-perceived health status repeat the sign and significance of the previous phase (with values of − 20.39% and − 24.55%, respectively). In this phase, male gender 11.52% and clinical diagnosis of lymphoma (Hodgkin or non-Hodgkin) (15.07%) vs. acute leukaemia, are all associated with an increased risk of financial catastrophe.

In the last phase of analysis, phase 3 (see Table 4) the number of statistically significant parameters associated with the independent variables is significantly lower. In this case, the separated/divorced marital status and good/very good self-perceived health status reduce the probability of catastrophe (by − 12.03% and − 42.18%, respectively), while the presence of multiple myeloma is the only variable that increases this probability.

## Discussion

In Europe, cancer treatment and medical costs are generally met by the public sector. However, other direct costs such as non-medical costs and time costs, together with indirect and psychosocial costs [74], are borne by patients and their families [75], and may represent a financial burden [76–78] that is so important [79, 80] that even when the disease has been overcome, the patient’s family may be forced into debt and/or asset reduction in order to provide continuing care [81].

For various medical conditions, informal care often plays a major role in addition to that of clinical treatment. Corroborating this, a recent review showed that in 2015 informal care accounted for 24.3% of the total costs for patients with cancer, representing a mean annual cost of 9927 euros [62]. The proportion of total costs ranged from 14% for patients with breast cancer [82] to 27% overall [83], rising to 33% for patients with colorectal cancer [84] and those in the final stage of life [85].

Various studies have analysed the financial effects arising from the out-of-pocket expenditure inherent to cancer. Most of these studies have focused on Asian countries, where public coverage of this type of expense is not usually generous, revealing that up to 40% of the families of cancer patients experience a catastrophic level of health expenditure [67–69, 86, 87]. Indeed, the financial pressure becomes such that some patients temporarily or permanently abandon the recommended treatment due to the impossibility of paying for it [70].

In our own study, the only sociodemographic factors related to a higher risk of such catastrophic consequences were found to be male gender (although this was only relevant in the second phase considered), aged less than 35 years and married marital status. This is consistent with the findings in the literature, according to which male gender increases the probability of a catastrophic financial outcome [67, 68]. However, in many studies this variable is either not statistically significant [71, 72] or the value of its coefficient is ambiguous, depending on the country where the study is conducted [69, 88], and therefore clear-cut conclusions about the effect of gender cannot be drawn.

Relative youth (age 35 years or younger) is only a significant factor in the risk of financial catastrophe with respect to the first phase (in the second and third, it is not statistically significant). This result contrasts with most previous findings [68, 69, 71, 72], among which only one study has found younger age to be a significant factor for financial catastrophe [67]. We believe the main reason for this discrepancy is that this segment of population presents low levels of income, either because they are young and unemployed, and depend entirely on relatives (usually parents), or because the income from employment during the first years of working life is low [89, 90].

In this context, married marital status was found to increase the risk. However, prior results are conflicting: only one study obtained the same result [69], while others concluded that married status was a protective factor against a catastrophic financial outcome for cancer patients and their families [68, 72]. We believe the negative impact of married status (as regards the financial implications of a change in care provision) can be explained as follows: in other marital statuses, there is a notably lower probability of access to informal care [56, 91] and so the elimination of this resource would not have such a dramatic effect on the financial situation of patients and their families. However, despite widows being less likely to receive informal care, an assessment of care needs is important and necessary, especially if they have no informal care and a monetary assessment of care cannot therefore be made.

Corroborating previous research findings, we show that a low level of education is a very important factor for financial catastrophe when informal care is replaced by formal care [67, 69, 72, 88], to a degree similar to that found for the loss of employment [68, 72]. In both cases, there is a direct connection between the variable in question and the likelihood that household income will be low. Accordingly, the household is more dependent on informal care and will have difficulty in accessing, or affording, any other type of care.

The self-perceived level of health is a very significant variable for receiving informal care [56, 92] and, therefore, a reliable predictor of financial catastrophe in the circumstances considered. This is also true of the diagnosis received. Thus, leukaemia (in the first two phases of analysis) and multiple myeloma (in the third) are associated with a greater risk of financial catastrophe if access to informal care is lost. The significance of these variables arises from the fact that chemotherapy is considerably more aggressive for leukaemia than for other types of cancer [56, 93], and therefore the side effects are more severe and the need for care is greater. On the other hand, the patients who survive usually achieve a full recovery of their previous quality of life [94]. In contrast, multiple myeloma has a very high long-term relapse rate (90%) [60] and a low probability of survival (35–37%) [95].

In Spain, an extraordinary home care service provided by the State and Autonomous Communities existed for those who needed care at home in an extraordinary or exceptional situation. This service was dependent on two aspects: 1) duration of the need for care was temporary and limited (not permanent), and 2) the family or closest relatives could not provide the care. However, this important service practically disappeared under the major legislative development in 2006, that is, the approval of Act 39/2006 of 14 December on the Promotion of Personal Autonomy and Assistance for Persons in a Situation of Dependency, also known as the Dependency Act [63]. The purpose of this Act was to address the long-term needs of persons who were unable to independently perform the basic activities of daily life. However, the act automatically excludes assessment and care for patients receiving chemotherapy or radiotherapy until this treatment has concluded [96], despite the fact that care needs are inevitably present prior to any transplant and during the first year post-transplant [56]. Therefore, in its basic design, this Act does not take into account the situation of cancer patients and that of their informal caregivers, who sacrifice their work and leisure time, and often suffer emotional stress and fatigue as acute as that produced by the treatment on the patients themselves. In this sense, we propose the incorporation of the previous extraordinary home care “temporary” service into the new regulatory framework of the Dependency Act, since the potential beneficiaries of extraordinary home care and Dependency Act are not the same.

On the other hand, social protection systems in Europe for the care of people are heterogeneous: while some countries rely on exclusive professional care, others have strong support for informal care. In this sense, a first recommendation would be to achieve a balance between the well-being of the people cared for and the caregivers. A second general recommendation, appropriate for Spain, is the need to achieve effective work-family reconciliation as regards care provision, especially in those countries where a large number of caregivers of working age are unemployed or are homemakers [97]. In the specific case of Spain, the design of social policies should focus on two main areas of action. The first should pay attention to family care, since this is a valuable resource in caring for people with needs derived from diseases or aging. The second should be oriented towards improving the well-being of the caregiver and necessitates a holistic approach that incorporates fiscal, assistance, regulatory and labour measures. Social service policies aimed at promoting the formation and strengthening of social support networks should also be included [98].

The study described in this paper has certain limitations. First, the sample only included patients with haematological neoplasia and who had received stem cell transplantation. This restriction was made to enable us to analyse the situation of informal caregiving not only during the chemotherapy phase, but also prior to the transplant and in the subsequent phases, which are often characterised by severe health complications. Secondly, the hypothesis of perfect and absolute substitution of all informal care by formal care is very severe. In practice, we believe the families concerned would acquire only the formal care believed to be indispensable, covering the patient’s most severe needs, thus minimising the financial impact on the household. In other words, we believe that formal care would replace certain specific tasks, beyond the reach of informal care [47], or tasks that inevitably require formal professional services, whilst in other cases the support of the informal caregiver would continue to be provided [99]. According to the literature, moreover, the optimal balance between informal and formal care depends on the type of disease, individual and household needs and the family situation, as well as the availability of public and professional resources. A third limitation is that the sociodemographic characteristics and the health profiles are limited in the present study, when the inclusion of the rather broadly differentiated variables would allow for the more accurate design of healthcare policies.

Future lines of research should be undertaken to extend the scope of the present study and to better characterise the sociodemographic and health profiles that are relevant to the current demand for informal care, in view of the fact that the foreseeable disappearance of this option would impose an enormous financial burden on the households affected. This type of research is necessary in the field of cancer in general, and for specific types of cancer in particular, as the future incidence of some cancers (such as those considered in this paper) is expected to increase. Finally, an analysis of the need for informal care should be included as an integral part of cost analyses in cancer studies, together with the direct health and non-health costs considered and indirect costs such as the loss of labour productivity.

## Conclusions

To the best of our knowledge, this is the first study to identify the sociodemographic and health factors associated with the catastrophic financial consequences of replacing informal care by formal care for persons with haematologic neoplasms.

Our findings highlight the need for legislators to design healthcare policies that include the aim of protecting patients with cancer from the risk of suffering an intolerable financial burden if informal care must be replaced by formal care. Therefore, specific sociodemographic and health profiles should be identified and taken into consideration. Among these characteristics, those that increase the probability of patients and their families suffering a catastrophic financial impact are married marital status, low education level, a self-perceived health status that is only fair or worse, and a diagnosis of leukaemia (in phase one or two, as defined above) or multiple myeloma (in phase three).

Before 2006, there existed a temporary and extraordinary home help service in Spain. Restoring this within the framework of the Dependency Act would improve the quality of life of patients with haematological neoplasm while reducing the workload of the informal caregiver.

## Data Availability

The datasets generated during and/or analysed during the current study are available from the corresponding author on reasonable request.

## Abbreviations

HSCT: Haematopoietic Stem Cell Transplantation
ICV: Informal care value

## References

[1] World Health Organization: Media centre. Cancer. 2017. Available at https://www.who.int/en/news-room/fact-sheets/detail/cancer. Accesed 31 Dec 2021.

[2] Spanish Society of Medical Oncology (2017). Cancer figures in Spain.

[3] Bray F, Ferlay J, Soerjomataram I, Siegel RL, Torre LA, Jemal A (2018). Global cancer statistics 2018: GLOBOCAN estimates of incidence and mortality worldwide for 36 cancers in 185 countries. CA Cancer J Clin.

[4] Ferlay J, Colombet M, Soerjomataram I, Dyba T, Randi G, Bettio M, Gavin A, Visser O, Bray F (2018). Cancer incidence and mortality patterns in Europe: estimates for 40 countries and 25 major cancers in 2018. Eur J Cancer.

[5] Economic and Social Council of Spain (2017). Memoria sobre la situación socioeconómica y laboral de España 2016.

[6] International Agency for Research on Cancer (2018). Global Cancer Observatory Globocan.

[7] Sommariva S, Pongiglione B, Tarricone R (2016). Impact of chemotherapy-induced nausea and vomiting on health-related quality of life and resource utilization: a systematic review. Crit Rev Oncol/Hematol.

[8] Schwartz A, Nail L, Chen R, Meek P, Barsevick A, King M, Jones L (2000). Fatique patterns observed in patients receiving chemotherapy and radiotherapy. Cancer Investig.

[9] Wu H-S, McSweeney M (2007). Cancer-related fatigue:“It's so much more than just being tired”. Eur J Oncol Nurs.

[10] Poole K, Froggatt K (2002). Loss of weight and loss of appetite in advanced cancer: a problem for the patient, the carer, or the health professional?. Palliat Med.

[11] Griffin A, Butow P, Coates A, Childs A, Ellis P, Dunn S, Tattersall M (1996). On the receiving end V: patient perceptions of the side effects of cancer chemotherapy in 1993. Ann Oncol.

[12] Portenoy RK, Lesage P (1999). Management of cancer pain. Lancet.

[13] Nikbakhsh N, Moudi S, Abbasian S, Khafri S (2014). Prevalence of depression and anxiety among cancer patients. Casp J Intern Med.

[14] Brown LF, Kroenke K (2009). Cancer-related fatigue and its associations with depression and anxiety: a systematic review. Psychosomatics.

[15] Yamauchi H, Nakagawa C, Fukuda T (2017). Social impacts of the work loss in cancer survivors. Breast Cancer.

[16] Romito F, Goldzweig G, Cormio C, Hagedoorn M, Andersen BL (2013). Informal caregiving for cancer patients. Cancer.

[17] Cooke L, Grant M, Eldredge DH, Maziarz RT, Nail LM (2011). Informal caregiving in hematopoietic blood and marrow transplant patients. Eur J Oncol Nurs.

[18] Prigerson HG, Bao Y, Shah MA, Paulk ME, LeBlanc TW, Schneider BJ, Garrido MM, Reid MC, Berlin DA, Adelson KB (2015). Chemotherapy use, performance status, and quality of life at the end of life. JAMA Oncol.

[19] Keohane EM, Otto CN, Walenga JM (2020). Rodak's hematology: clinical principles and applications.

[20] McCarthy PL, Hahn T, Hassebroek A, Bredeson C, Gajewski J, Hale G, Isola L, Lazarus HM, Lee SJ, LeMaistre CF (2013). Trends in use of and survival after autologous hematopoietic cell transplantation in North America, 1995-2005: significant improvement in survival for lymphoma and myeloma during a period of increasing recipient age. Biol Blood Marrow Transplant.

[21] Jayani R, Rosko A, Olin R, Artz A (2020). Use of geriatric assessment in hematopoietic cell transplant. J Geriatr Oncol.

[22] Bevans MF, Mitchell SA, Marden S (2008). The symptom experience in the first 100 days following allogeneic hematopoietic stem cell transplantation (HSCT). Support Care Cancer.

[23] Ferlay J, Soerjomataram I, Ervik M, Dikshit R, Eser S, Mathers C, Rebelo M, Parkin D, Forman D, Bray F (2013). GLOBOCAN 2012 v1. 0, Cancer incidence and mortality worldwide: IARC CancerBase no. 11 [internet].

[24] Mourad N, Michel RP, Marcus VA (2019). Pathology of gastrointestinal and liver complications of hematopoietic stem cell transplantation. Arch Pathol Lab Med.

[25] Kurosawa S, Oshima K, Yamaguchi T, Yanagisawa A, Fukuda T, Kanamori H, Mori T, Takahashi S, Kondo T, Kohno A (2017). Quality of life after allogeneic hematopoietic cell transplantation according to affected organ and severity of chronic graft-versus-host disease. Biol Blood Marrow Transplant.

[26] Li J, Song Y, Gu D, Dupre ME (2019). Formal and informal care. Encyclopedia of gerontology and population aging.

[27] Riedel M, Kraus M (2011). The Organisation of Formal Long-Term Care for the Elderly. Results from the 21 European Country Studies in the ANCIEN Project. ENEPRI Research Report No 95.

[28] Del Pozo-Rubio R, Jiménez-Rubio D (2019). Catastrophic risk associated with out-of-pocket payments for long term care in Spain. Health Policy.

[29] Van den Berg B, Brouwer WB, Koopmanschap MA (2004). Economic valuation of informal care. Eur J Health Econ.

[30] McCrone P, Allcock LM, Burn DJ (2007). Predicting the cost of Parkinson's disease. Mov Disord.

[31] World Health Organization. Lessons for long-term care policy: the cross-cluster initiative on long-term care. Geneva: World Health Organization; 2002.

[32] Torvinen S, Färkkilä N, Roine RP, Sintonen H, Saarto T, Taari K (2016). Costs in different states of prostate cancer. Acta Oncol.

[33] Jakobsen M, Poulsen PB, Reiche T, Nissen NP, Gundgaard J (2011). Costs of informal care for people suffering from dementia: evidence from a Danish survey. Dement Geriatr Cogn Disord Extra.

[34] König H-H, Leicht H, Brettschneider C, Bachmann C, Bickel H, Fuchs A, Jessen F, Köhler M, Luppa M, Mösch E (2014). The costs of dementia from the societal perspective: is care provided in the community really cheaper than nursing home care?. J Am Med Dir Assoc.

[35] Oleen-Burkey M, Castelli-Haley J, Lage MJ, Johnson KP (2012). Burden of a multiple sclerosis relapse. Patient.

[36] Rao GN, Bharath S (2013). Cost of dementia care in India: delusion or reality?. Indian J Public Health.

[37] Kim Y, Given BA (2008). Quality of life of family caregivers of cancer survivors. Cancer.

[38] Girgis A, Lambert S, Johnson C, Waller A, Currow D (2012). Physical, psychosocial, relationship, and economic burden of caring for people with cancer: a review. J Oncol Pract.

[39] Kim Y, Spillers RL, Hall DL (2012). Quality of life of family caregivers 5 years after a relative's cancer diagnosis: follow-up of the national quality of life survey for caregivers. Psycho-Oncol.

[40] Kim Y, Schulz R (2008). Family caregivers' strains: comparative analysis of cancer caregiving with dementia, diabetes, and frail elderly caregiving. J Aging Health.

[41] Goren A, Gilloteau I, Lees M, daCosta DiBonaventura M (2014). Quantifying the burden of informal caregiving for patients with cancer in Europe. Support Care Cancer.

[42] Tan JY, Molassiotis A, Lloyd-Williams M, Yorke J (2018). Burden, emotional distress and quality of life among informal caregivers of lung cancer patients: an exploratory study. Eur J Cancer Care.

[43] Maguire R, Hanly P, Hyland P, Sharp L (2018). Understanding burden in caregivers of colorectal cancer survivors: what role do patient and caregiver factors play?. Eur J Cancer Care.

[44] Stenberg U, Ruland CM, Miaskowski C (2010). Review of the literature on the effects of caring for a patient with cancer. Psycho-Oncol.

[45] Davey A, Patsios D (1999). Formal and informal community care to older adults: comparative analysis of the United States and Great Britain. J Fam Econ Iss.

[46] Rogero-García J (2009). Distribución en España del cuidado formal e informal a las personas de 65 y más años en situación de dependencia. Rev Esp Salud Publica.

[47] Jiménez-Martín S, Vilaplana-Prieto C (2012). The trade-off between formal and informal care in Spain. Eur J Health Econ.

[48] Keating N, Fast J, Forbes D, Wenger C. Informal care networks of Canadian seniors with long-term health problems. Health Canada. Ottawa: National Health Research and Development Program; 2002.

[49] OECD (2017). Health at Glance.

[50] Mestheneos E, Triantafillou J. Supporting family carers of older people in Europe-the Pan-European background report. Siglo del Hombre Editores. 2005;

[51] European Commission. The 2015 Ageing Report: Economic and budgetary projections for the 28 EU Member States (2013-2060). Directorate-General for Econ Financ Affairs Econ Policy Committee (AWG). 3(2015):142–162

[52] Carrera F, Pavolini E, Ranci C, Sabbatini A. Long-term care systems in comparative perspective: care needs, informal and formal coverage, and social impacts in European countries. Reforms in long-term care policies in Europe. New York: Springer; 2013. p. 23–52.

[53] Fujisawa R, Colombo F. The long-term care workforce: overview and strategies to adapt supply to a growing demand. OECD Health Working PaPers. 2009;1:1–63.

[54] Fernández, J.L., Forder, J., Trukeschitz, B., Rokosová, M., McDaid, D. (World Health Organization): How can European states design efficient, equitable and sustainable funding systems for long-term care for older people (2009).

[55] Vickland V, Werner J, Morris T, McDonnell G, Draper B, Low L-F, Brodaty H (2011). Who pays and who benefits? How different models of shared responsibilities between formal and informal carers influence projections of costs of dementia management. BMC Public Health.

[56] Ortega-Ortega M, Montero-Granados R, Romero-Aguilar A (2015). Sociodemographic and clinical factors associated with informal Care in Hematologic Malignancy Patients: a study based on different phases of the treatment. Rev Esp Salud Publica.

[57] Ream E, Pedersen V, Oakley C, Richardson A, Taylor C, Verity R (2013). Informal carers' experiences and needs when supporting patients through chemotherapy: a mixed method study. Eur J Cancer Care.

[58] Ortega-Ortega M, del Pozo-Rubio R (2019). Catastrophic financial effect of replacing informal care with formal care: a study based on haematological neoplasms. Eur J Health Econ.

[59] Ortega-Ortega M (2015). Healthcare costs and non-healthcare costs associated with the treatment to haematologic cancer patients: an economic perspective. Doctoral Dissertation University of Granada Spain.

[60] Carreras E, Martinez C (2010). Manual de trasplante hematopoyético. Ed Antares 4°Ed.

[61] Ortega-Ortega M, Montero-Granados R, de Dios Jiménez-Aguilera J (2018). Differences in the economic valuation and determining factors of informal care over time: the case of blood cancer. Gacet Sanit.

[62] Oliva-Moreno J, Trapero-Bertran M, Peña-Longobardo LM, del Pozo-Rubio R (2017). The valuation of informal Care in Cost-of-Illness Studies: a systematic review. Pharmacoecon.

[63] Official Bulletin State (2006). Act 39/2006 of 14th December on Promotion of Personal Autonomy and Assistance for Persons in a Situation of Dependency.

[64] Wagstaff A, van Doorslaer E (2003). Catastrophe and impoverishment in paying for health care: with applications to Vietnam 1993-1998. Health Econ.

[65] Tomini SM, Packard TG, Tomini F (2012). Catastrophic and impoverishing effects of out-of-pocket payments for health care in Albania: evidence from Albania living standards measurement surveys 2002, 2005 and 2008. Health Policy Plan.

[66] Stiglitz JE (2000). Economics of the public sector. WW Norton & Company Ltd.

[67] Leng A, Jing J, Nicholas S, Wang J (2019). Catastrophic health expenditure of cancer patients at the end-of-life: a retrospective observational study in China. BMC Palliat Care.

[68] Lee M, Yoon K (2019). Catastrophic health expenditures and its inequality in households with Cancer patients: a panel study. Processes.

[69] Lee M, Yoon K, Choi M (2018). Private health insurance and catastrophic health expenditures of households with cancer patients in South Korea. Eur J Cancer Care.

[70] Kaisaeng N, Harpe SE, Carroll NV (2014). Out-of-pocket costs and oral cancer medication discontinuation in the elderly. J Manag Care Spec Pharm.

[71] Hoang VM, Pham CP, Vu QM, Ngo TT, Tran DH, Bui D, et al. Household financial burden and poverty impacts of Cancer treatment in Vietnam. Biomed Res Int. 2017;8 10.1155/2017/9350147 PMC5585616 28904976

[72] Group TAS (2015). Catastrophic health expenditure and 12-month mortality associated with cancer in Southeast Asia: results from a longitudinal study in eight countries. BMC Med.

[73] Fahrmeir L, Kneib T, Lang S, Marx B (2013). Regression: models, methods and applications.

[74] Brown ML, Yabroff KR, Schottenfeld D, Faumeni J (2006). Economic impact of Cancer in the United States. Cancer epidemiology and prevention.

[75] Pisu M, Azuero A, McNees P, Burkhardt J, Benz R, Meneses K (2010). The out of pocket cost of breast cancer survivors: a review. J Cancer Surviv.

[76] Chen AB, Li L, Cronin AM, Brooks GA, Kavanagh BD, Schrag D (2018). Estimating costs of care attributable to cancer: does the choice of comparison group matter?. Health Serv Res.

[77] Bestvina CM, Zullig LL, Zafar SY (2014). The implications of out-of-pocket cost of cancer treatment in the USA: a critical appraisal of the literature. Future Oncol.

[78] Azzani M, Roslani AC, Su TT (2015). The perceived cancer-related financial hardship among patients and their families: a systematic review. Support Care Cancer.

[79] Altice CK, Banegas MP, Tucker-Seeley RD, Yabroff KR. Financial hardships experienced by cancer survivors: a systematic review. J Natl Cancer Inst. 2017;109 10.1093/jnci/djw205 PMC6075571 27754926

[80] Hayman JA, Langa KM, Kabeto MU, Katz SJ, DeMonner SM, Chernew ME, Slavin MB, Fendrick AM (2001). Estimating the cost of informal caregiving for elderly patients with cancer. J Clin Oncol.

[81] Skalla KA, Smith EML, Li Z, Gates C. Multidimensional needs of caregivers for patients with cancer. Clin J Oncol Nurs. 2013;17 10.1188/13.CJON.17-05AP 23956004

[82] Gordon L, Scuffham P, Hayes S, Newman B (2007). Exploring the economic impact of breast cancers during the 18 months following diagnosis. Psycho-Oncol.

[83] Marti J, Hall PS, Hamilton P, Hulme CT, Jones H, Velikova G, Ashley L, Wright P (2016). The economic burden of cancer in the UK: a study of survivors treated with curative intent. Psycho-Oncol.

[84] Färkkilä N, Torvinen S, Sintonen H, Saarto T, Järvinen H, Hänninen J, Taari K, Roine RP (2015). Costs of colorectal cancer in different states of the disease. Acta Oncol.

[85] Round J, Jones L, Morris S (2015). Estimating the cost of caring for people with cancer at the end of life: a modelling study. Palliat Med.

[86] Choi JW, Cho KH, Choi Y, Han KT, Kwon JA, Park EC (2014). Changes in economic status of households associated with catastrophic health expenditures for cancer in South Korea. Asian Pac J Cancer Prev.

[87] Aryankhesal A, Etemadi M, Mohseni M, Azami-Aghdash S, Nakhaei M (2018). Catastrophic health expenditure in Iran: a review article. Iran J Public Health.

[88] Arsenijevic J, Pavlova M, Rechel B, Groot W (2016). Catastrophic health care expenditure among older people with chronic diseases in 15 European countries. PLoS One.

[89] Ben-Porath Y (1967). The production of human capital and the life cycle of earnings. J Political Econ.

[90] Heckman JJ (1976). A life-cycle model of earnings, learning, and consumption. J Political Econ.

[91] Yabroff KR, Kim Y (2009). Time costs associated with informal caregiving for cancer survivors. Cancer.

[92] Mor V, Allen SM, Siegel K, Houts P (1992). Determinants of need and unmet need among cancer patients residing at home. Health Serv Res.

[93] De Linares Fernández S, Contreras Molina C, Fernández Cordero I (2007). Guía informativa trasplante de médula ósea.

[94] Redaelli A, Stephens JM, Brandt S, Botteman MF, Pashos CL (2014). Short-and long-term effects of acute myeloid leukemia on patient health-related quality of life. Cancer Treat Rev.

[95] Kristinsson SY, Landgren O, Dickman PW, Derolf AR, Bjorkholm M (2007). Patterns of survival in multiple myeloma: a population-based study of patients diagnosed in Sweden from 1973 to 2003. J Clin Oncol.

[96] Official Bulletin State. Real Decreto 174/2011, de 11 de febrero, por el que se aprueba el baremo de valoración de la situación de dependencia establecido por la Ley 39/2006, de 14 de diciembre, de Promoción de la Autonomía Personal y Atención a las personas en situación de dependencia (2011).

[97] Peña-Longobardo LM, Oliva-Moreno J. The economic value of non-professional care: a Europe-wide analysis. Int J Health Policy Manag :1–15. 2021; 10.34172/ijhpm.2021.149 PMC9808255 34814681

[98] García-Mochón L, Peña-Longobardo LM, del Río-Lozano M, Oliva-Moreno J, Larrañaga-Padill I, García-Calvente MDM (2019). Determinants of burden and satisfaction in informal caregivers: two sides of the same coin? The CUIDAR-SE study. Int J Environ Res Public Health.

[99] Chappell N, Blandford A (1991). Informal and formal care: exploring the complementarity. Ageing Soc.

